# National strategy for the integration of pharmacovigilance in the Moroccan TB Control Program

**DOI:** 10.11604/pamj.2017.26.48.7394

**Published:** 2017-01-31

**Authors:** Driss Soussi Tanani, Samira Serragui, Sanae Hammi, Latifa Ait Moussa, Abdelmajid Soulaymani, Rachida Soulaymani, Yahia Cherrah

**Affiliations:** 1 Department of Pharmacology, Faculty of Medicine and Pharmacy, University of Abdelmalek Essaadi Tanger 90100, Morocco; 2 Department of Pharmacology and Toxicology, Faculty of Medicine and Pharmacy, University of Mohamed V Rabat 10170, Morocco; 3 Department of Pneumology, Faculty of Medicine and Pharmacy, University of Abdelmalek Essaadi Tanger 90100, Morocco; 4 Moroccan Anti Poison and Pharmacovigilance Center, Rabat 10170, Morocco; 5 Laboratory of Genetics and Biometry, University Ibn Tofail, Kenitra 14000, Morocco

**Keywords:** Pharmacovigilance, adverse drug reactions, antituberculosis drugs

## Abstract

The objective of this work is to demonstrate the interest of integration of pharmacovigilance in Moroccan Tuberculosis Control Program (MTCP). The integration of pharmacovigilance in MTCP was conducted in October 2012with the Global Fund support. We compared the reports notified before and after this integration (period 1: January 2010–October2012; period 2: October 2012–December 2013). The detection of signals was based on the Information Component available inVigiMine. We used the SPSS version 10.0 and Med Calc version 7.3 for data analysis. The average number of spontaneous reports increased from 3.6 to 37.4 cases/month (P< 10^-3^). The average age was 40.7 ± 17.5 years; the sex ratio was 0.8. Hepatic reactions (32.7%) predominated during the first period, while skin reactions (24.1%) were in the second period (P = 10^-4^), and40.9% of cases in the first period were serious against 15.8% in second period (P = 0.003). Nine signals were generated (hepaticenzyme increase, cholestasis, jaundice, arthralgia, acne, lower limb edema, pruritus, skin rashes, and vomiting). The integration of pharmacovigilance in Moroccan Tuberculosis Control Program improved the management of ADRs and detected new signals of antituberculosis drugs.

## Introduction

The use of medicines is an important aspect of many public health programmes (PHPs) that are designed to improve the health of a target population. Their cost to the health budget is between 6% in developed countries and 45% in some developing countries, but there are huge variations between both developed countries and developing countries [1]. Medicines are important for treating and preventing diseases, but the success of PHPs is closely linked to the availability of effective and safe medicines and patient confidence in these PHPs.

Despite the progress that has been made in pharmacovigilance (PV), the scourge on PHPs of adverse drug reactions(ADRs) remains significant. Pharmacoeconomic studies on the costs of ADRs suggest that governments pay considerable amounts from their health budgets towards covering the costs associated with them. In a meta-analysis of 39 prospective studies from hospitals in the United States, it was shown that ADRs ranked from the fourth to sixth leading cause of death [2].

A prospective study in England to the whole National Health Service bed base, suggested that for patients aged > 16 years, at any onetime the equivalent of up to seven 800-bed hospitals may be occupied by patients admitted with ADRs [3]. WHO has produced guidelines for setting up a national PV centre [4] and many WHO PHPs have developed their own guidelines.

With increasing population coverage, the chances of developing adverse reactions and interactions will increase, as programmes are extended to the more vulnerable populations such as the young, the elderly, pregnant women and people with malnutrition and disease. In addition, health practitioners and the public need more information about the potential benefit, rationality of use and risk of the medicines given. National TB programmes are generally well struc¬tured to treat patients by using standardized indicators, but they do not collect enough information on ADRs to optimize the treatment of tuberculosis discarding preventable ADRs.

Tuberculosis in Morocco remains also a public health problem with an average incidence of 83.5 cases per 105 inhabitants in 2011. Resistant TB form represents 1.3% of the whole cases. As public health programmes (PHPs) are extended to the more vulnerable populations such as the young, the elderly, pregnant women, and people with malnutrition, the chances of developing ADRs and interactions will increase. In addition, health practitioners and the public need more information about the potential benefit, rationality of use, and risk of the medicines given.

Morocco has an efficient PV system through its national PV center (NPVC) that is a member WHO collaborating for Francophone African countries and Middle Eastern countries since 2011. Recently there have been some initiatives within countries or under the leadership of WHO, to create and develop subsystems for pharmacovigilance (PV) to monitor the specific products used in their PHPs. Morocco is among the first countries which received grants from the Global Fund to strengthen PV in AIDS and TB [5].

The objective of this work is to demonstrate the various steps of successful model of integrating PV in Moroccan Tuberculosis Control Program (PV-MTCP).

## Methods

The integration of PV in the MTCP was held with the support of the Department of Epidemiology and the Global Fund. A day of launch of this integration was held on October 11, 2012 in the presence of WHO representative in Morocco, the majority of physicians responsible for the treatment of TB in MTCP and some responsible of Department of Epidemiology. The aim of this day was focused on the interests of PV in PHPs, particularly MTCP for rational use of TB drugs and patient safety. After this day of large sensitization on the PV, successive actions were carried out for health professionals working on TB in Morocco and for health professionals working in the PV center.

### Actions for TB health professionals

*Sessions of sensitization:* we conducted several sessions of intensive PV to report anti-TB induced ADRs for all TB health practitioners of 16 Moroccan regions. These formations were performed in the presence of three teams: pharmacovigilance team: its objective was focused on the importance of spontaneous reporting of all TB ADRs by answering the following questions: How to report? What to report? When to report? Who should report?; university hospital of phthisiology team: its objective was to elucidate the sequence of TB treatment protocols with minimal risk, and to show their expertise in the management of TB ADRs; epidemiology team: its objective was to validate an administrative system to route notifications from declaring to PV center.

*Registers of notification:* are notebooks that contain 50 yellow cards triplicate notification so that each team keeps a copy of traceabilitythrough the new system of reporting.

*Designation of regional referring physicians in PV:* for each region of the kingdom, a physician referring of PV was designed with the aim of offrire sensitization stunts for medical and paramedical staff, and solve the problems of spontaneous reports.

**Figure 1:**
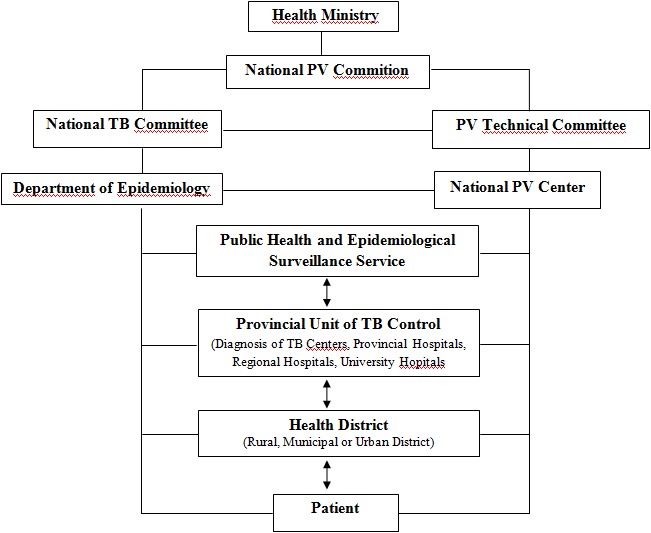
National Reporting System of anti-TB ADRs

*Validation of a reporting system:* after some tests from spontaneous reports via the new reporting system, some faults occurred impeding the flow of notifications to the PV center. Then all the referring physicians of regions were invited to the PV center with the three teams to solve various problems observed and definitively validate an efficient national reporting system (Figure 1).

### Actions made by PV health professionals

*Designation of national coordinator of PV in MTCP:* a pharmacologist physician of NPVC was designated the first day of the PV-MTCP integration to ensure the good functioning of all processes of reporting, to be the national referring of anti-TB drugs PV, to promote spontaneous reporting and detect eventual signals. Therefore, the coordinator conducted an internship in Bordeaux for automatic signal detection.

*New technical committee of PV:* this committee was formally restructured with new members from different specialties to discuss possible problems caused by drugs in general and especially in TB.

*Collection of reports:* the NPVC accepts all anti-TB drugs events arriving through this reporting system at different stages of the diagram. If a problem is blocking notification of ADR at any stage of the diagram, this one is sent to the next stage until it reaches the NPVC. The NPVC accepts all means of notifications (fax, postal mail, email, web site, phone, watt sap)to facilitate the reporting process.

*Relationship and causality assessment:* each reported case is analyzed methodically using the following approach: pathophysiological study of the form of the disease; pharmacological study of drug suspects; study the cause and effect relationship: both French method [6, 7] and WHO method [8] are using; analysis of the national and international database is performed on the reported effect to estimate its frequency, and if the effect is rarely more research is conducted.

*Feed back to reporters:* once the research is sufficient, a scientific answer based on the medical evidence is sent to the reporter for convince and retain him to the reporting.

*Vigi-TB:* the national coordinator of PV established an electronic registry of ADRs reporting that contains all the email addresses of pneumo-phthisiologist physicians and their staff phone. It serves as a mean to provide a feedback to the network of MTDC practitioners, to follow up their continual participation and to provide them a monthly line listing concerning all reported ADRs and a ranking of the top three of the MTCP reporters is sent to everyone. Academic healthcare professionals also receive answers of the NPVC and are also involved in the exchange of information.

*Data processing managing:* all reported cases are stored in a local database managed by the coordinator of PV in MTCP and are sent to the international database in Uppsala Monitoring Center (UMC) by Vigi flow software [9] and Vigi Mine software detects international signals [10].

*Signal management:* the NPVC followed the methodology of European Medicines Agency to detect and manage signals [11].

The definition of signal is: “Information that arises from one or multiple sources (incl. observations and experiments), which suggest a new potentially causal association, or a new aspect of a known association, between an intervention and an event or set of related events, either adverse or beneficial, that is judged to be of sufficient likelihood to justify verificatory action” [12]. The signal management steps are the following.

Signal detection include: qualitative signal detection by analysis of case-by-case of individual case reports in our local database; quantitative signal detection utilises statistical methods to identify frequent combinations of a drug and an event that occur with disproportionately high frequency in large spontaneous report databases.

The NPVC use 3statistical methods: the Proportional Reporting Ratio (PRR) [13], the Reporting Odds Ratio (ROR) [14] to detect signals in Moroccan database and Information Component (IC) [9] to detect signals in international database (VigiMine).

Signal validation: by a scientific committee ofPV, composed of pharmaco-toxicologists and medical experts in the field of TB, who studiesnumber of reports, temporal association, plausible mechanism, de/rechallenge, severity, novelty, possible drug-drug interactions, special populations in which the reaction occurs. The committee also studies other relevant sources of information from larger data sets. A signal becomes validated, if all relevant documentation is suggestive of a new causal association, or a new aspect of known association, and therefore justifies further assessment.

Signal prioritization and assessment: the team of PV studies: impact on patients: severity, reversibility, consequences of treatment; important public health impact; effect on benefit-risk balance; pharmacological, medical and epidemiological assessment; study of preventability; strengths and limitations of data used for signal generation, need for additional data; a need to conduct a cohort study to confirm causality and highlight the risk factors of signal.

Implementation of risk minimization measures: after demonstrating the impact of the signal on patients and on the MTCP, risk minimization measures are implemented to limit the physical and moral damage.

Exchange of information: by signals on data sharing, collection of additional data, more accurate assessment, facilitation of decision making.

**Study design and setting:** IT was a retrospective study (January 1, 2010–October 11, 2012) with a passive PV and without Global Fund support, compared with a prospective study (October 11, 2012–December 2013), conducted in all Moroccan TB diagnosis centers (135 MTDC) which are in charge of treating TB patients, after integrating PV-MTCP. These MTDC exist in all cities and receive about 27000 TB patients per year. The comparison between the 2 periods concernedthe number of reports, methods used for collecting ADRs(spontaneous reporting in period 1 versus intensive PVreporting in period 2), nature, and seriousness of ADRs.

**Study population:** Patients treated for TB and only who reported one or more ADRs were included in this study. TB patients who did not present ADRs were excluded from this study.

**Analysis:** The data were collected using Microsoft Excel software (version 5.1), and variables were described as percentage or mean (±SD). Qualitative data were compared using Chi-2 test or Fisher exact test. Quantitative data were compared using Student’s t-test. The statistical significance level was set at P< 0.05. Analysis was performed using SPSS (version 10.0) and MedCalc (version 7.3) Software.

**Pharmacovigilance monitoring indicators:** The NPVC used PV monitoring indicators for the evaluation of this integration: *Structural indicators:* the existence of national regulations for PV; the existence of a budget for this activity; the availability of staff working full time in PV; the existence of a PV cases management system; the availability of means of communication to the NPVC; the existence of a PV technical committee; the existence of a national commission of PV.

*Process indicators:* are tools that directly or indirectly assess the functionality of the system: main indicator: Number of reported cases per month (TB patients). Specific indicators: number of signals generated; realization of a risk management plan.

*Impact indicators:* to evaluate the effectiveness of the system to achieve its goal: number of serious cases between the 2 periods; regularly sending cases to Uppsala Monitoring Centre (UMC); number of works done in this integration.

## Results

The international database during the globalperiod of study (January 2010–December 2013) showed thatMorocco with an average TB incidence (50–100 cases/105inhabitants) has recorded 927 ADRs (Table 1).

**Table 1:** International database VigiSearch (January 2010- December 2013)

Country	Number of reports	TB Incidence (/10^5^)
Republic of Korea	4650	100-300
India	2960	100-300
USA	1076	<24
Morocco	927	50-100
France	302	<24
South Africa	191	>300
Russian Federation	70	100-300
Tunisia	85	<24

### Descriptive study: general information

608 cases were reported (927 ADRs) during the study (January 2010–December 2013). The average age of patients was 40.7 ±17.5 years with a sex ratio of 0.8. The most prescribed anti-TB drug was ERIP-K4 (83.7%) since it contains 4combined anti-TB drugs (Ethambutol, Rifampicin, Isoniazid, and Pyrazinamide) followed by Riniazide 10.1% (Rifampicin, Isoniazid) as maintenance treatment. Accountability of cases according to the WHO method showed that 5% had a certain relationship of cause to effect, 15% had a probable relationship and 80% had a possible relationship. 140 of the cases were serious (23%) with 8 deaths (1.3%), 21 cases had a commitment prognosis (3.4%), 4 cases developed sequelae (0.6%), and 107 cases required hospitalization or prolongation of hospitalization (17.5%). The outcome was favorable in 47.2% of cases, 16.2% were healing, 35.2 were unknown, and 1.3% died. For fatal cases, there were 3 women aged between 20and 29 years: TB MDR with lower limb edema, lymphnode TB in a pregnant woman with fulminant hepatitis, and pulmonary TB with toxic epidermal necrolysis; there were5 men aged between 28 and 70 years: TB MDR with lowerlimb edema, pulmonary TB with hepatic encephalopathy, multifocal TB with cytolytic hepatitis, pulmonary TB with cholestatic hepatitis, and TB + heart failure with fulminant hepatitis (Table 2).

**Table 2:** Caracteristics of eight died cases

Age (year)	Sexe	Indication	ADR	Accountability
20	F	Multiresistant TB	Lower limb edema	Possible
30	M	Multiresistant TB	Lower limb edema	Possible
28	M	Pulmonary TB	Hepatic	Probable
70	M	Multifocal TB	encephalopathy	Possible
44	M	Pulmonary TB	Cytolytic hepatitis	Possible
-	M	TB + Heart failure	Cholestatic hepatitis	Possible
29	F	Lymph node TB+	Hepatitis	Probable
27	F	Pregnancy	Fulminant hepatitis	Possible
		Pulmonary TB	Lyell syndrome	

### Descriptive study: nature of ADRs (Table 3)

The majority of ADRs occurred during the first month after starting treatment with a difference depending on the nature of the ADRs (Graph 1). ADRs of liver and biliary system disorders(32.8%) predominated in period 1 with cytolytic hepatitisas the most predominant symptom followed by skin and appendages disorders (26.3%) with pruritus as the most predominant symptom, while ADRs of skin and appendages’disorders predominated in period 2 (24.2%) followed byADRs gastrointestinal system disorders (21%) with epigastric pain and vomiting as the most predominant symptom (Table 3).

**Table 3:** Comparaison of ADRs types before and after integration of PV-MTCP

System Organ Class (Disorders)	Period 1 n(%)	Period 2 n(%)	P
Skin and appendages disorders	86 (26.3)	145 (24.2)	NS
Gastro-intestinal system disorders	29 (8.9)	126 (21)	0.007
Liver and biliary system disorders	107 (32.8)	87 (14.5)	NS
General disorders	12 (3.7)	86 (14.4)	0.0007
Central and peripheral nervous	42 (12.9)	46 (7.7)	NS
system	8 (2.4)	36 (6)	0.03
Musculo-skeletal system disorders	6 (1.8)	20 (3.4)	0.04
Psychiatric disorders	0	10 (1.7)	-
Hearing and vestibular disorders	0	10 (1.7)	-
Respiratory system disorders	21 (6.4)	9 (1.5)	0.02
Metabolic disorders	5 (1.5)	6 (1)	NS
Platelet, bleeding and clotting	0	6 (1)	-
disorders	0	5 (0.8)	-
Endocrine disorders	2 (0.6)	3 (0.5)	NS
Vision disorders	2 (0.6)	2 (0.3)	NS
Heart rate and rhythm disorders	2 (0.6)	1 (0.1)	NS
White cell disorders	5 (1.5)	1 (0.1)	NS
Red blood cell disorders	0	1 (0.1)	-
Urinary system disorders	**327 (100)**	**600 (100)**	
Reproductive disorders			
**TOTAL**			

NS: Not significant

### Analytic study

We compared the notification before and after PV-MTCP. The average number of reports increased from 3.6 to 37.4 cases/month (P< 10^-3^). The System Organ Class of ADRs reported during the first period concerned mainly liver and biliary system disorders (32.8%) because prescribers were reporting mainly serious ADRs, while skin and appendage system disorders (24.2%) of ADRs were predominantly reported in the second period. New ADRs occurred in period 2 as ADRs of hearing and vestibular system disorders, respiratory system disorders, endocrine system disorders, visual system disorders, and reproductive system disorders (Table 3). The comparison of seriousness cases before and after integration of PV-MTCP showed that in period 1 there were more hospitalization (28.9%) and development life threatening (9.6%) (Table 4). The comparison of ADRs outcome before and after integration of PV-MTCP showed that in period 2 the outcome of ADRs was more favourable with less unknown cases but without significant difference (Table 5).

**Table 4:** Comparaison of seriousness cases before and after integration of PV-MTCP

Seriousness P	Period 1 n (%)	Period 2 n (%)
Hospitalization/prolonged <0,001	51(28.9)	56(13)
Life-threatening <0,001	17(9.6)	4(1)
Sequelae	2(1.2)	2(0.4)
Death	NS	
NS	2(1.2)	6(1.4)
**Total 72(40,9)**	**68(15.8)**	**<0,001**

NS: Not significant

**Table 5:** Comparaison of cases evolution before and after integration of PV-MTCP

Evolution	Period 1 (%)	Period 2 (%)	P
Favorable	41.8	52.7	NS
Ongoing	15.6	16.9	NS
Unknown	41.4	29	NS
Lethality	1.2	1.4	
NS			
Total	100	100	

NS: Not significant

Signals detection The signal detection was focused on the cases related to combined anti-TB form (ERIPK4) recorded in VigiMine. We found 875 ADRs related to this combination; 268 of them were issued from Morocco(30.6%). 18 international signals have been generated; 11 of them were from Morocco (Table 6).

**Table 6:** Moroccan signals with anti-TB combined form

Nature of Signal	International IC_025_	Moroccan IC_025_
Hepatitis+	3.69	0.12
Increase hepatic enzymes	2.85	2.79
Jaundice	2.68	1.25
Cholestatic hepatitis+	2.16	1
Acne	1.54	0.67
Arthralgia	1.54	2.54
Vomiting	1.33	0.76
Pruritus	1.14	1.78
Abdominal pain	0.60	0.33
Periperal neuropathy+	0.41	0.18
Peripheral edema	0.14	1.98

+ Critical ADRs signal

## Discussion

The international database (Vigiflow) during the period of study (January 2010–December 2013) showed that Morocco with an average TB incidence (50-100 cases/10^5^ inhabitants) has recorded 608 cases (927 ADRs). 176 cases were reported (327 ADRs) before PV-MTCP (January 2010–October 2012)and 432 cases were reported (600 ADRs) after PV-MTCP(October 2012–December 2013). USA is the best notifier country (1076 ADRs) with a low incidence (<24/10 ^5^ inhabitants) and South Africa is the worst notifier country (191ADRs) with the high incidence (>300/10^5^ inhabitants). However, Republic of Korea and India have the highest rate of notifications (4650 and 2960 ADRs, resp.) due to their high incidence (100-300 cases/10^5^ inhabitants), Table 1. This increase of ADRs in period 2 was due to the effective integration of PV in the MTCP with awareness of the majority of anti-TB prescribers and involvement for spontaneous reporting of TB ADRs and feedback with practical procedures of ADRs management are regularly sent to anti-TB prescribers to motivate them to reporting ADRs. In our study, females had a higher incidence of ADRs. In general, females are at a higher risk of developing ADRs [15]. It might be because they pass through life stages like pregnancy, menarche, and so forth, which modify the drug response [16]. Studies from UK and Canada also reported females to have a significantly higher incidence of ADRs due to anti-TB drugs [17, 18]. This suggests the need for special precautions while prescribing anti-TB drugs to females.

**Figure 2:**
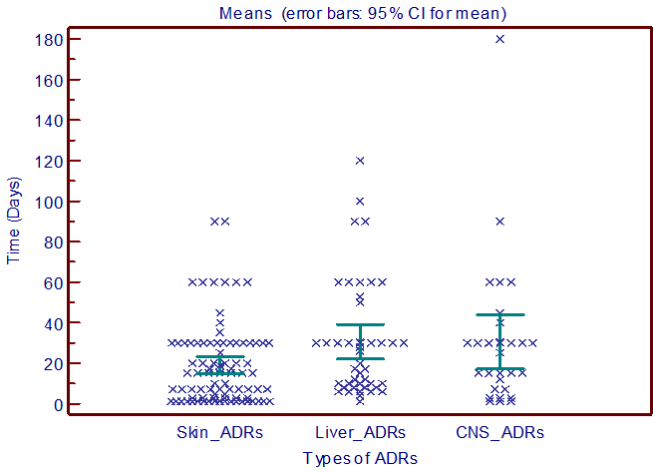
Comparison of average times to onset of cutaneous, hepatic and neurological ADRs

We compared the average time of onset of cutaneous, hepatic, and neurological ADRs. Cutaneous ADRs appeared first with an average time of 18.9 ± 20.0 days. Hepatic and neurological ADRs are with almost identical average time (30.5 ± 28.4 and 30.4 ± 36.0 days) but with a wide standard deviation for neurological ADRs (Figure 2). Cutaneous ADRs appeared first because their mechanism is often immune allergic against hepatic ADRs and neurological ADRs take more time to appear. The majority of ADRs occurred during the first month after starting treatment requiring more vigilance during this period regardless of the nature of ADRs. Onset of the ADRs is an important factor helpful in early detection of the ADRs. Also in studies from India [19] and from Nepal [20] more than half of ADRs occurred within the first 30 days after starting TB treatment. It is essential for the healthcare professionals to counsel the patients regarding the early identification of ADRs in the first few weeks. Regular monitoring of the patients during these initial weeks might be essential for early detection of ADRs. On the severity of ADRs, cases of period 2 were significantlyless severe than period 1 (15.8% versus 40.9%, P<0.001) with less hospitalization (13% versus 28.9%, P< 0.001)and less development life-threatening (1% versus 9.6%, P<0.001). Eight patients died (1.3%) with an average age of36.8 ± 18.0 years and a male sex ratio = 1.6, among which5 died by hepatic complications, 2 had a multiresistant TB, and one died by Lyell syndrome (Table 2). Twenty-one cases(3.4%) had ADRs development life-threatening, and 4 cases developed sequelae 0.6% (2 left deafness, 2 ataxo-spasmodic diseases), Table 4.

In Morocco, the average of TB incidence has stagnated during recent years, about 81 cases per 100000 inhabitants [21]; then the increase of ADRs during period 2 is mainly due to the integration PV-MTCP. The reporters during the first period were in the majority from university and provincial hospitals, but in the second period they were mainly from MTDC. Among the 16 regions of Morocco, 7 were involved in the reporting of ADRs in the first period, whereas 14 regions were engaged in the second period. The System Organ Class of ADRs reported during the first period concerned mainly liver and biliary system disorders because the physicians reported mainly serious ADRs as hepatitis, while skin and appendage system disorders of ADRs were predominantly reported in the second period. This second period is quite rich in ADRs because it has been active PV which forced prescribers to notify all ADRs. There was a significant increase of reports in gastrointestinal, general, musculoskeletal, and psychiatric system disorders between2 periods but a significant decrease of reports in metabolic system disorders because the notifications during the first period emanated mainly from university hospitals (Table 3). There were new ADRs of reproductive, vision, respiratory, hearing, and vestibular and endocrine disorders notified in the second period after PV-MTCP reflecting the interest of active PV.

The comparison of ADRs evolution before and after integration of PV-MTCP showed that in period 2 the outcome of ADRs was more favorable (52.7% versus 41.8%) with less unknown cases (29% versus 41.4%) but without significant difference (Table 5).

The most common system affected by the ADRs in our study (PV-MTCP) was skin and appendage (24.2%). Also in2 studies from Thailand and Malaysia, skin and appendage system was the most affected (48.9 and 49.5%) [22, 23]. In an Indian study, the majority of the patients (53%) had gastrointestinal reactions [19]. In 2 studies from Nepal and Iran [20, 24], the most common system affected by the ADRs was liver and biliary system (58.5 and 37%). The principal clinical risk factors for hepatotoxicity are old age, malnutrition, alcoholism, HIV infection, and chronic hepatitis B and C infections [25]. There are several strategies to prevent the occurrence of these ADRs. Drug induced hepatic dysfunction usually occurs within the initial few weeks of the intensive phase of anti-TB chemotherapy [25]. It is also recommended that liver function should be studied every two weeks during ATT to prevent serious hepatotoxicity [26]. A few guidelines were also published mentioning the management of hepatotoxicity due to anti-TB drugs [27, 28]. It is also the responsibility of the healthcare professionals to counsel the patients regarding the early signs of hepatotoxicity.

For minimizing risk of serious ADRs, the MPVC collaborating with some hepatologists and phthisiologists developed a practical procedure of TB hepatotoxicity that helps prescribers to manage the risks associated with anti-TB drugs. Adverse drug reactions to certain drugs may differ within each country, reflecting different patterns of prescription, socioeconomic status, and culture.

On December 31, 2013, 18 international signals have been generated with combined anti-TB form (ERIP-K4). Among these 18 signals, 11 were from Morocco including 3 critical signals: hepatitis, cholestatic hepatitis, and peripheral neuropathy. Three signals had a Moroccan score of IC_025_ higher than international score of IC_025_: arthralgia, pruritus, and peripheral oedema, testifying to the importance of these three signals (Table 6).

All these Moroccan signals are known except peripheral edema including lower limb edema which is a new signal not documented in the literature. Therefore, a technical committee of PV met in July 2013 to discuss these signals. Recommendations were issued for increased vigilance of these signals especially lower limb edema requiring more laboratory investigations to rule out other causes of the occurrence of edema. The committee decided also to initiate a study to evaluate the relationship of accountability of this significant lower limb edema.

The NPVC used PV monitoring indicators of WHO [1] for the evaluation of this integration. As structural indicators, there was a budget offered by the global funding for this activity for the monitoring of adverse reactions to TB drugs and the availability of staff working full time in PV that manage the activity with the national reporting system and Vigi-TB. The existence of a new PV technical committee and a national commission of PV strengthened the management of this activity.

**Figure 3:**
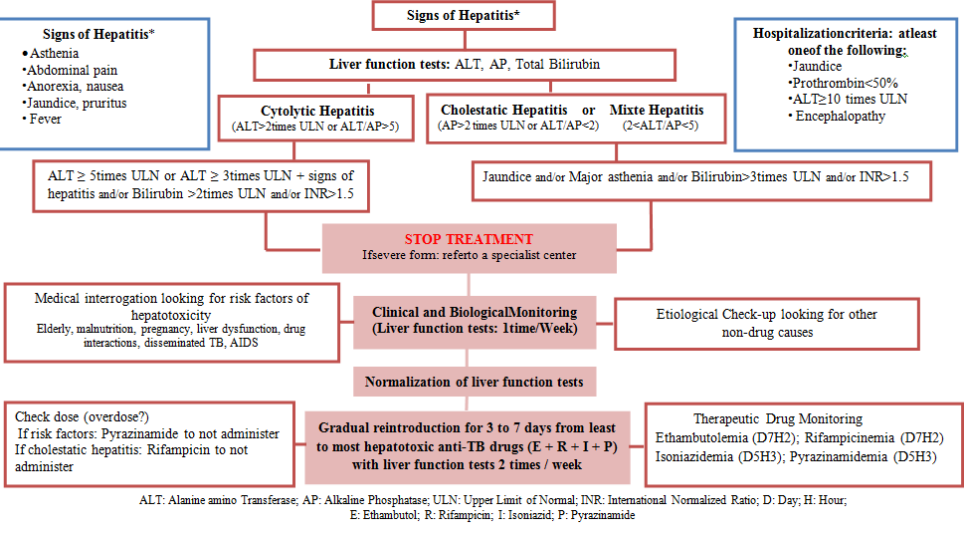
Management of anti-TB drugs induced hepatotoxicity in Morocco

As Process indicators, the average number of reports increased from 3.6 to 37.4 cases/month (P<10^-3^) after integration of PV-MTCP. Eleven signals were generated from Morocco including 3critical signals (hepatitis, cholestatic hepatitis, and peripheral neuropathy) with a new signal the lower limb edema never documented in the literature. As hepatotoxicity is common and a leading cause of mortality in our study, we carried out a risk minimization plan with the collaboration of experts and the plan was distributed to all health professionals to prevent and manage hepatotoxicity (Figure 3).

As impact indicators, on the severity of ADRs, cases of period 2 were significantly less severe than period 1 (15.8% versus 40.9%, P<0.001) with less hospitalization (13% versus 28.9%, P<0.001) and less development life-threatening (1% versus 9.6%, P<0.001). There was no significant difference in deaths between period 1 and 2 (1.2%, 1.4%). All cases were regularly sent to Uppsala Monitoring Centre (UMC)allowing the generation of Moroccan signals by VigiMine.

Many works have been done after this integration, we quote: Oral communication: Pharmacovigilance of antituberculosis drugs in Morocco(1st International Congress of Pharmacology May 2012, Rabat Morocco); Oral communication: The antituberculosis adverse drug reactions: The Moroccan experience (ISOP November 2012, Cancun Mexico); Poster : Pharmacovigilance in Moroccan Tuberculosis Control Programme(ISOP October 2013, Pisa Italy); Oral communication: New signal: Lower limb edema following use of the ERIP-K4 (1st African Congress of Pharmacovigilance, December 2013, Rabat Morocco); Article: Pharmacovigilance and Moroccan Tuberculosis Public Program: current situation. (Tuberculosis Research and Treatment. 2014); Poster: Risk Management of Anti-TB Drugs Induced Liver Injury in Morocco (ISOP October 2014, Tianjin China); Poster: New Signal Management: Lower limb edema induced by ERIP-K4 (ISOP October 2014, Tianjin China); Article: Signal management of disproportionate reporting of lower limb edema induced by anti-tuberculosis drugs in Morocco(Journal of Pharmacovigilance 2015); Poster: Metabolic side effects following the use of anti-TB drugs in Morocco(ISOP October2015, PragueCzech Republic); Oral communication: Activity report of Integrating Pharmacovigilance in Moroccan Tuberculosis Control Programme.

## Limitations

The strong point of our study is the collection of all major and minor ADRs, but the limitation is the absence of files of patients who have not developed ADRs for estimating the incidence of ADRs and risk factors.

## Conclusion

This work demonstrated that the integration of pharmacovigilance is crucial to the success of any public health program using medicines. Pharmacovigilance should be an integral part of every public health program to optimize the use of scarce health resources and prevent potential tragedies.
